# Identification of long-term trends and seasonality in high-frequency water quality data from the Yangtze River basin, China

**DOI:** 10.1371/journal.pone.0188889

**Published:** 2018-02-21

**Authors:** Weili Duan, Bin He, Yaning Chen, Shan Zou, Yi Wang, Daniel Nover, Wen Chen, Guishan Yang

**Affiliations:** 1 State Key Laboratory of Desert and Oasis Ecology, Xinjiang Institute of Ecology and Geography, Chinese Academy of Sciences (CAS), Urumqi, Xinjiang, China; 2 Key Laboratory of Watershed Geographic Sciences, Nanjing Institute of Geography and Limnology, Chinese Academy of Sciences, Nanjing, China; 3 Hengyang Normal University, Hengyang, China; 4 Department of Engineering, University of California–Merced, United States of America; Universidade de Aveiro, PORTUGAL

## Abstract

Comprehensive understanding of the long-term trends and seasonality of water quality is important for controlling water pollution. This study focuses on spatio-temporal distributions, long-term trends, and seasonality of water quality in the Yangtze River basin using a combination of the seasonal Mann-Kendall test and time-series decomposition. The used weekly water quality data were from 17 environmental stations for the period January 2004 to December 2015. Results show gradual improvement in water quality during this period in the Yangtze River basin and greater improvement in the Uppermost Yangtze River basin. The larger cities, with high GDP and population density, experienced relatively higher pollution levels due to discharge of industrial and household wastewater. There are higher pollution levels in Xiang and Gan River basins, as indicated by higher NH_4_-N and COD_Mn_ concentrations measured at the stations within these basins. Significant trends in water quality were identified for the 2004–2015 period. Operations of the three Gorges Reservoir (TGR) enhanced pH fluctuations and possibly attenuated COD_Mn_, and NH_4_-N transportation. Finally, seasonal cycles of varying strength were detected for time-series of pollutants in river discharge. Seasonal patterns in pH indicate that maxima appear in winter, and minima in summer, with the opposite true for COD_Mn_. Accurate understanding of long-term trends and seasonality are necessary goals of water quality monitoring system efforts and the analysis methods described here provide essential information for effectively controlling water pollution.

## Introduction

Since 1979, China has experienced sustained and rapid economic growth with a concurrent and growing problem of environmental pollution [1–3]. With rapid urban development and accelerating industrialization, the demand for water is increasing, and meanwhile water quality is declining because of pollution. All these changes have made water security a major contemporary challenge [4, 5]. According to the “2015 China Environmental Bulletin” in June 2016 [6], the proportion of water quality stations belonging to the best water quality category in seven major river basins (the Yangtze River, the Yellow River, the Pearl River, the Huai River, the Hai River, the Songhua River and the Liao River) in 2014 is 64.5%. There is no obvious change from 2014 to 2016, and much work will be necessary to achieve the 70% target (the ratio of the best water quality category in seven major river basins) for water quality by 2020. Pollution control and water quality restoration in these river basins are therefore key for future environmental efforts.

In order to make rivers and lakes clean and prevent water from being polluted, a surface water quality monitoring system was built by China's Ministry of Environmental Protection in 2004. This system is similar to the National Water-Quality Assessment (NAWQA) Program in the United States [7, 8], the Harmonized Monitoring Scheme (HMS) in Britain [9, 10], and the National Monitoring and Assessment Program (NOVA) in Denmark [11, 12]. These systems can obtain reliable water quality data, which is the first step to estimate and control the water pollution. Meanwhile, based on these monitoring data, trend analysis (e.g., linear trend test and Mann–Kendall trend test [13]), load estimation methods (e.g., LOADEST[14]), and evaluation models (e.g., SPARROW[15], SWAT[16]) for water quality evaluation have been gradually developed and improved [17], and all these methods have been used successfully to assess pollution at a basin scale [18]. However, using a combination of several methods, few academic studies have employed these datasets to improve water quality assessments and regional water resources management by analyzing numerous parameters over various spatio-temporal scales in China [19, 20].

The Yangtze River plays a significant role in the history, culture, and economy of China, and it discharges large amounts of sediment and freshwater into the East China Sea, thus influencing the sea’s biogeochemical cycles. The aquatic environment of the Yangtze has been degraded drastically during recent decades [21, 22]. Current research on Yangtze River water quality monitoring focuses on the evaluation of water quality for human health, regional water pollution characteristics [23, 24] and related modeling exercises [25]. However, based on weekly data, little research has been done to analyze and understand seasonal water quality trends on the Yangtze River over ten years.

The objectives of this study are therefore (1) to analyze spatial and temporal distributions of water quality data collected from January 2004 to December 2015 at 17 monitoring stations in the Yangtze River basin, (2) to calculate trends and observe seasonality, and (3) to explore the relationship between water quality and river discharge, using a combination of several trend analysis methods including the seasonal Mann-Kendall test and time-series decomposition.

## Data and methods

### Study area

**Fig 1:**
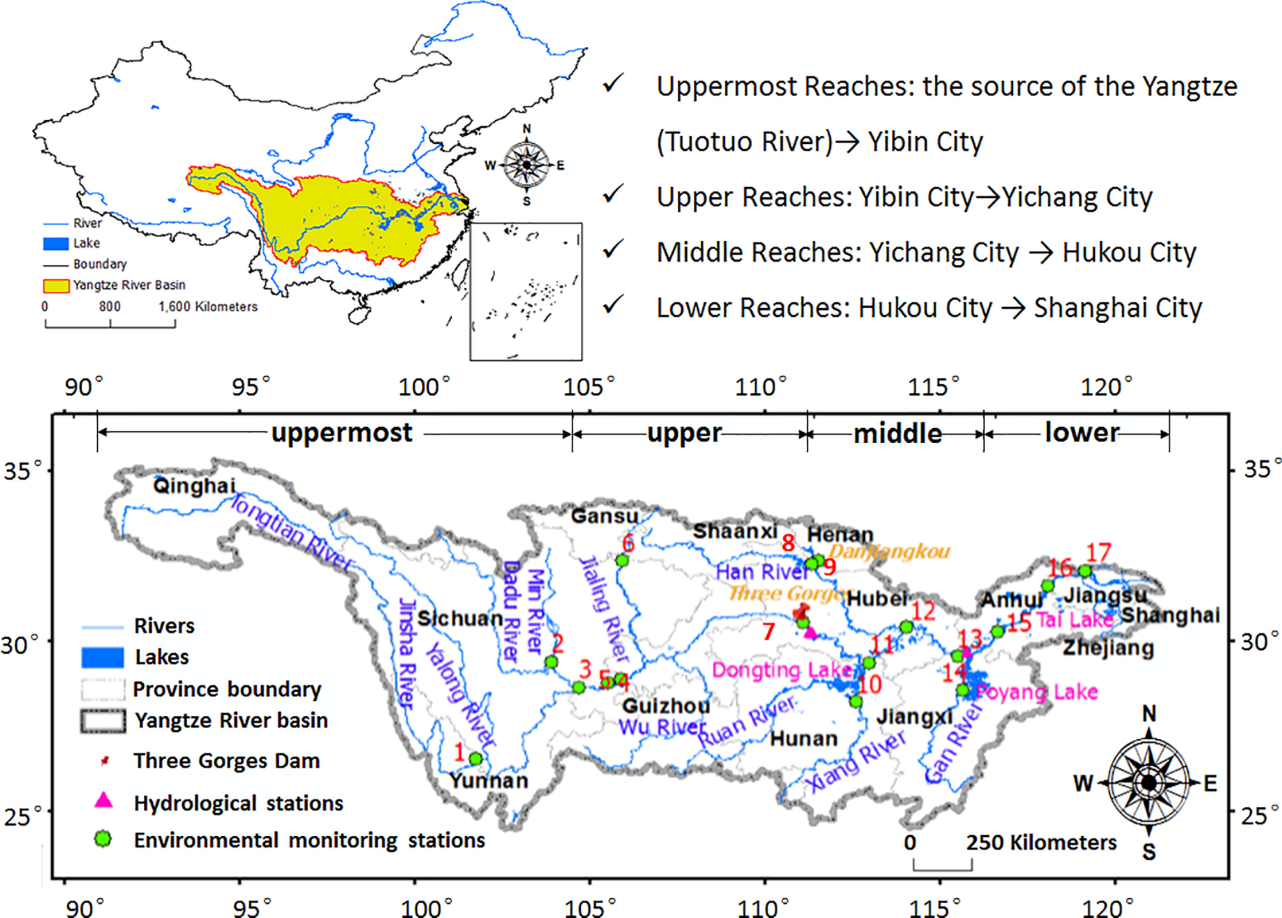
Geological map of the Yangtze River basin and studying stations.

The Yangtze River, originates from Tanggula Mountain (elev. 6621 m), and flows 6,397 km eastward before emptying into the Eastern China Sea (ESC). Its drainage basin lies between 91°E and 122°E and 25°N and 35°N, covering one fifth of the whole nation with an area of about 1.8 ×10^6^ km^2^ (Fig 1). The river is divided into an uppermost section (from the source to Yibin, Sichuan Province, with a length of 3,499 km), an upper section (from Yibin to Yichang, Hubei Province, with a length of 1,030 km), a middle section (from Yichang to Hukou, Jiangxi Province, with a length of 950 km) and a lower section (from Hukou to Shanghai, with a length of 938 km). The main river meanders through diverse landforms from west to east. The whole basin has complicated hydroclimatic conditions because of the East and South Asian monsoon activities, which brings seasonal discharge from wet (May to October) to dry (December to April) conditions. The mean annual precipitation varies from 300 to 500 mm in the western region to 1,600–1,900 mm in the southeastern region, and the annual mean temperature in the southern and northern parts of the middle and lower Yangtze River basin is 19 and 15°C, respectively [26]. The Yangtze River basin supplies about 50% of the country’s runoff and 40% of Chinese freshwater resources [27].

### Datasets

A total of 17 environmental monitoring stations were selected for analysis in this study, in which 7 stations (Number 1, 5, 7, 11, 13, 15 and 16) are located along the trunk stream, 2 stations (8 and 9) were setup for monitoring the Danjiangkou Reservoir, and the rest were built to assess the influence of major tributaries (see Fig 1 and Table 1). Weekly water quality data (including pH, COD_Mn_, NH_4_-N, and DO) at these 17 stations were obtained and processed from a surface water quality monitoring system. The monitoring system covers seven river systems including the Yangtze, the Yellow, the Pearl, the Huai, the Hai, the Songhua and the Liao Rivers. So far, 148 monitoring stations have been completely established, containing 122 stations for monitoring rivers and 26 stations for monitoring lakes. All these stations test five water quality parameters including pH, total organic carbon (TOC), chemical oxygen demand (COD_Mn_), ammonia-nitrogen (NH_4_-N), and dissolved oxygen (DO), every four hours. These data are processed by the government and used to generate weekly data and then the weekly data are published through the Data Center of China's Ministry of Environmental Protection. So only weekly pH, COD_Mn_, NH_4_-N, and DO were selected and because generally the water quality data are not made public in China. Data were collected at all stations for the period 2004–2015, except for three stations (Qingfengxia, Xingang and Chuchuo) which were only available for 2006 to 2015. Weekly water grades are also described according to environmental quality standards for surface waters in China (GB3838-2002, see S1 Table) (Grade I-V level means that water is “Excellent”, “Good”, “Satisfactory”, “Bad”, and “Very bad” respectively).

**Table 1:** Location and description of 17 environmental monitoring stations.

Number	Name	Province	River	Longitude	Latitude	Time period
1	Londong	Sichuang	Yangtze River	101.5064	26.59738	2004–2015
2	Minjiang Bride	Sichuang	Min River	103.7679	29.57433	
3	Liangjianggou	Sichuang	Min River	104.6	28.78333	
4	Tuojiang Bridge	Sichuang	Tuo River	105.4333	28.9	
5	Zhutuo	Chongqing	Yangtze River	105.849	29.01533	
6	Qingfengxia	Sichuang	Jianglin River	105.8786	32.66583	2006–2015
7	Nanjingguan	Hubei	Yangtze River	111.2707	30.76381	2004–2015
8	Taofen	Henan	Danjiangkou Reservoir	111.7053	32.66917	
9	Hujialing	Hubei	Danjiangkou Reservoir	111.5022	32.56639	
10	Xingang	Hunan	Xiang River	112.8378	28.34111	2006–2015
11	Chenglinji	Hunan	Yangtze River	113.2261	29.5404	2004–2015
12	Zongguan	Hubei	Han River	114.3333	30.61667	
13	Hexishuichang	Jiangxi	Yangtze River	115.847	29.74	
14	Chuchuo	Jiangxi	Gan River	116.1167	29.51667	2006–2015
15	Wanhekou	Anhui	Yangtze River	117.0261	30.50472	2004–2015
16	Linshan	Jiangsu	Yangtze River	118.5239	31.88639	
17	Snajiangying	Jiangsu	Jia River	119.6542	32.3513	

Notes: Here, the Yangtze River represents the trunk stream, seven stations are located on the trunk stream. Other rivers are the major tributaries.

Data related to river discharge, land use, gross domestic product (GDP), population density, and precipitation in the Yangtze River basin were also collected in order to analyze causes of spatiotemporal changes of water quality. Two hydrological stations (Yichang and Hukou), located in the junction zone of the upper, the middle and the lower Yangtze River, were chosen to obtain daily runoff data (Fig 1). The Yichang hydrological station is next to the Nanjingguan environmental station, and the Hukou hydrological station is next to the Hexishuichang environmental station. Land use data from 2010 were extracted from Landsat TM/ETM images with a spatial resolution of 1 km × 1 km at the national scale [28] and land use types were categorized as forest, wetland, desert, agriculture, grass, settlement, and others. Population and GDP for 2010 were provided by the Data Center for Resources and Environmental Sciences, Chinese Academy of Sciences (RESDC), with 1km×1km resolution. Average annual precipitation from 2000 to 2009 came from the China meteorological data center.

### Methods

#### Data pre-processing and summary statistics

After the data extraction process, weekly water quality data and daily runoff data were categorized and transformed into an Excel-format database. MATLAB (R2010a) was used to identify and assess data quality [29]. Data were then analyzed using descriptive statistics to quantitatively describe the main features of the dataset and time-series analysis (such as box plots) was performed on weekly data (the 'weekly' data come from processing of samples taken at 4 hourly time-steps) to allow a direct comparison between the hydrochemical information at different stations. Summary statistics including mean, median, and standard deviation were calculated for each pollutant. Box plots were used to evaluate changes in water quality and ArcGIS 10.0 was applied to display the spatial and temporal features of water quality at different stations. In addition, the Pearson's correlation coefficient [30] was used to evaluate the relationship between water quality and river discharge.

To further understand water quality variability for the whole Yangtze River basin, the full period 2004–2015 was divided into three sub-periods (2004–2007, 2008–2011, and 2012–2015), which can obtain more characters for the changes of water quality. We fisrt checked the trend for the whole period and then the probability distribution functions (PDFs) were calculated for regional mean (the average value of water quality at 17 stations) pH, COD_Mn_, NH_4_-N, and DO concentrations for all three sub-periods. A two-tailed Kolmogorov-Smirnov test was applied to assess whether the probabilities for different time periods are significantly different.

#### Seasonal Mann–Kendall trend analysis

The seasonal Mann–Kendall test (SMK) proposed by [31] was employed to detect monotonic trends of weekly water quality data. It is a nonparametric test and is used to detect potential trend change points in water quality trends [32–35]. Let *X* = (*X*_1_,*X*_2_,…,*X*_*n*_)^*T*^ be a time series of independent water quality observations, and *X*_*i*_ = (*X*_*i*1_,*X*_*i*2_,…,*X*_*ij*_). Here, *n* is the number of years and *j* is the number of weekly data for each year and so for weekly ‘seasons’, the first week data are compared only with the first weeks data of every year, the second week data only with the second weeks data of every year, and so on. The null hypothesis (*H*_0_) is that there are no monotonic trends in time. The statistic for the *gth* season is:

$$S_{g}=\sum_{i=1}^{n-1}\sum_{j=i+1}^{n}sgn(X_{jg}-X_{ig}),g=1,2,\ldots ,m$$ (1)

According to Hirsch et al. (1982), the SMK statistic, $\hat{S}$, for the entire series is calculated according to

$$\hat{S}=\sum_{g=1}^{m}S_{g}$$ (2)

For detailed treatment of this analysis, see [31]. Here, the significance level *p* is selected at 0.05 and 0.10 with corresponding *Z* statistics of 1.96 and 1.65, respectively. A positive value of *Z* indicates an 'upward trend' and a negative value of *Z* indicates a 'downward trend'.

#### Time-series decomposition

Analysis of long-term trends and seasonal variability was carried out using the dynamic harmonic regression (DHR) technique, extensively described in [36]. Compared to a modified Clausius–Clapeyron equation, a multiple linear regression that includes both a linear and a harmonic dependence on time, and digital filtration (DF), DRH better fits the data and captures both the seasonal variations of the pollutant concentrations and the smaller scale interannual variations in the long term trends [37]. This method has been employed successfully in many examples with non-stationary environmental time-series analysis [38–40]. DRH decomposes an observed time series into component parts such as trend and seasonality [41]

$$y_{t}=T_{t}+C_{t}+S_{t}+e_{t}$$ (3)

where *y*_*t*_ is the water quality time series, *T*_*t*_ is a longer-term trend or low frequency component), *C*_*t*_ is a sustained cyclical component with a period separate from the seasonal component (e.g. a diurnal cycle), *S*_*t*_ is the seasonal component (e.g., annual water quality seasonality), and *e*_*t*_ is the residuals for water quality predications.

The trend (*T*_*t*_) was extracted by the integrated random walk (IRW) model, which is a special case of the generalized random walk (GRW) [42]. The seasonal components (*S*_*t*_) were defined and calculated as follows:

$$S_{t}=\sum_{i}^{N/2}[a_{i,t}\cos\left(\omega_{i}t\right)+b_{i,t}\sin\left(\omega_{i}t\right)]$$ (4)

$$\omega_{i}=\frac{2\pi ∙i}{N}\;i=1,2,\cdots ,.[\frac{N}{2}]$$ (5)

where *ω*_*i*_ values are the fundamental and harmonic frequencies associated with the periodicity in the observed water quality series chosen by reference to the spectral properties. For instance, the period 52 corresponds to a weekly sampling data in an annual cycle. The generalized random walk was applied to model the phase and amplitude parameters, which were estimated recursively using the Kalman filter and a fixed interval smoother. The squared correlation coefficient between the calculated seasonal component and detrended water quality data was used to determine significance of seasonality; the significance of the trend was decided on the basis of the squared correlation coefficient between the calculated trend and deseasonalized water quality data. Finally, the coefficient of determination (*R*^2^) was applied to evaluate the performance of the model.

$$R^{2}=1-\frac{var(residuals)}{var(observations)}$$ (6)

where *var* is the variance estimate of the analyzed weekly water quality series.

Here, considering the regional representativeness of stations in different river segments and the distance between hydrological stations and environmental stations, two environmental stations (Nanjinguan and Hexishuichang) and two hydrological stations (Yichang and Hukou) that represent a large fraction of the total data were selected to present and discuss the DHR analysis. The runoff at Yichang station was highly impacted by the TGD, but it may also reflect the changes in annual runoff. The runoff data are daily data. Before discussing the correlations between runoff and water quality, we calculated the monthly annual runoff and water quality.

## Results

### Overall water quality and temporal/spatial distribution

**Fig 2:**
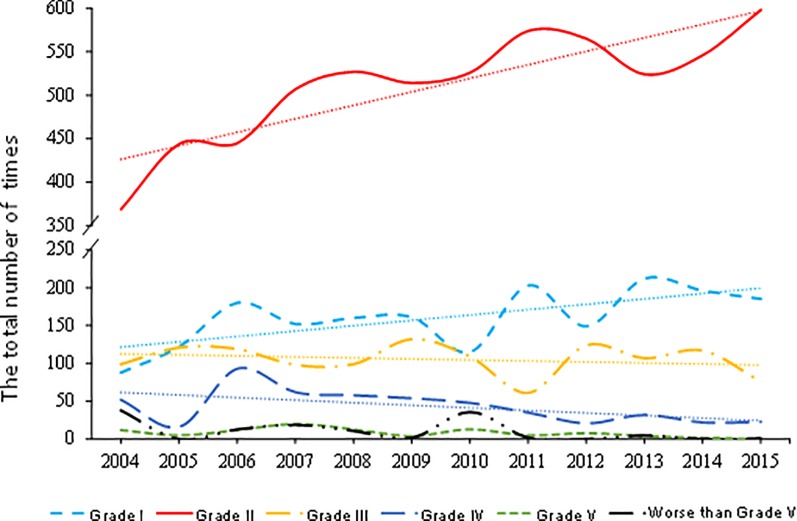
Changes of number of different grades of river water based on 17 stations in the Yangtze River basin from 2004 to 2015. The dashed lines are the trend lines for each Grade.

**Fig 3:**
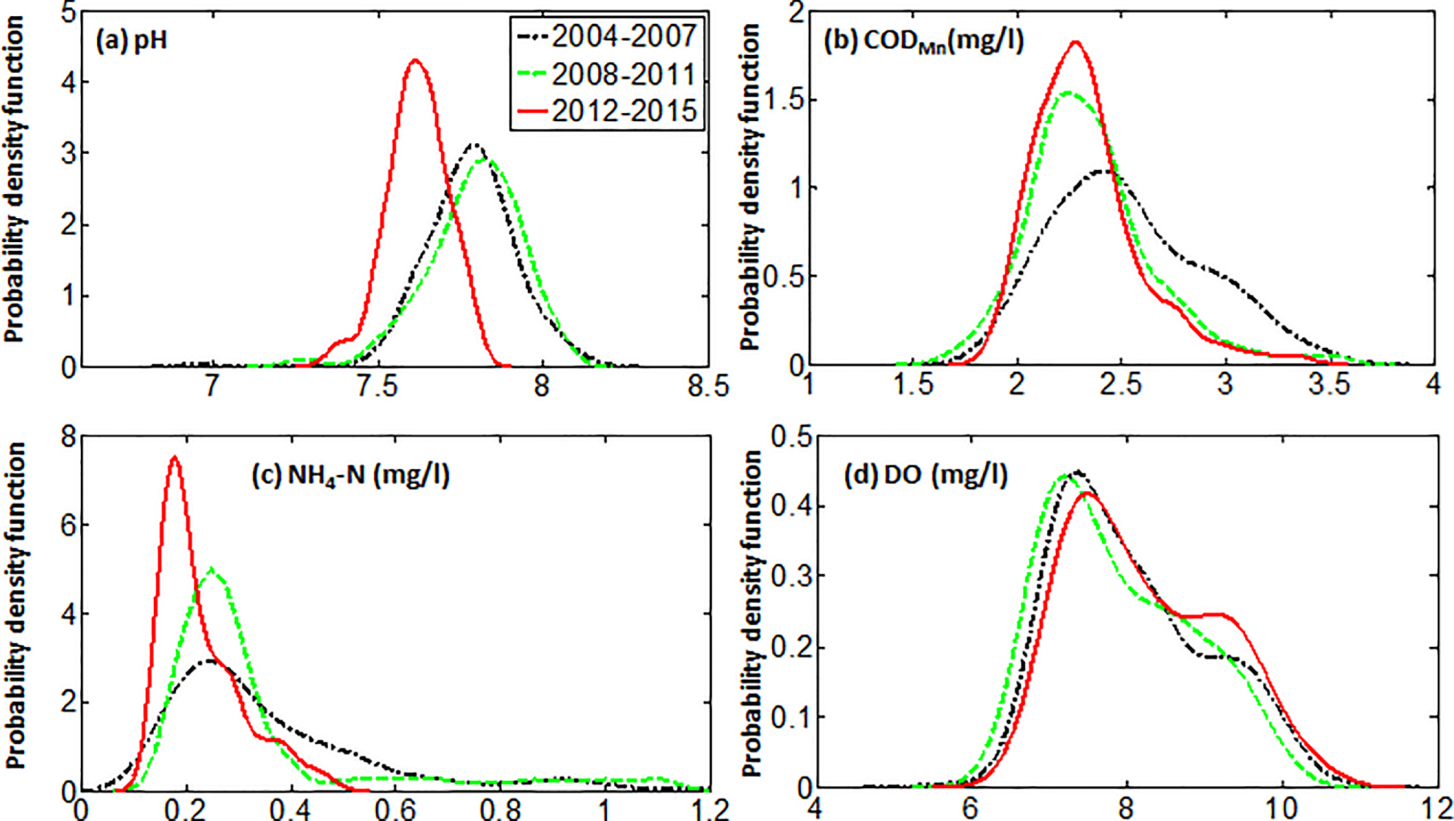
Weekly probability distribution functions for regional mean concentrations in the Yangtze River basin, between 2004 and 2015 for the three time periods: 2004–2007 (black curve), 2008–2011 (green curve), and 2012–2015 (red curve). (a) pH, (**b**) COD_Mn_ (mg/L), (**c**) NH_3_-N (mg/L), and (**d**) DO (mg/L).

Analysis shows that overall water quality improved from 2004 to 2015 (Figs 2 and 3), consistent with the results from [19]. Fig 2 shows changes in different grades of river water at 17 stations in the Yangtze River basin from 2004 to 2015 and shows a marked increase in Grade II water, occupying 66.7% of all water quality categories in 2015, and a slight increase in Grade I water, and a slight decrease in water of Grades III-IV. S1 Fig indicates overall change in fraction of Grade I-III water, Grade IV-V water, and Grade V+ water. Proportion of Grade I-III water has increased from 2004 to 2015, with 97.3% in 2015, suggesting gradual improvement in water quality in this time period.

The univariate statistics of the water quality parameters are presented in Table 2, and the distribution of pH, DO, COD_Mn_, and NH_4_-N from 2004 to 2015 at 17 stations are shown in the boxplots in S2 Fig. Generally, as the figure shows, the median pH and DO concentration at Station 1 and 6 are generally higher relative to the other stations, while the median COD_Mn_ and NH_3_-N concentrations are lower, revealing that the water quality in the Uppermost Yangtze River basin is better than in other areas. Except for Station 1, we see increasing concentrations of median DO and pH from Station 2 to Station 7, but decreasing concentrations of median COD_Mn_ and NH_4_-N concentrations. Station 2 (Minjiang Bride) had the highest median COD_Mn_ concentration (3.47 mg/l), the lowest median DO concentration (6.47 mg/l), and relative highly NH_4_-N concentration. We also found that the water was generally below Grade III during this period, consistent with previous work demonstrating that the middle and lower Min River was seriously polluted during 2003–2008 [43]. Station 14 (Chuchuo) had the highest median NH_4_-N concentration (0.97 mg/l) and the lowest median pH (6.94), followed by station 10 (Xingang), revealing high ammonia nitrogen pollution in the Xiang River and Gan River.

**Table 2:** Univariate statistics of the water quality parameters.

Station	pH		DO		COD_Mn_		NH_4_-N	
	Mean	SD	Mean	SD	Mean	SD	Mean	SD
1	8.21	0.33	8.48	1.41	2.29	2.52	0.51	1.84
2	7.57	0.28	6.50	1.94	3.47	1.11	0.50	0.50
3	7.74	0.46	8.29	1.64	2.49	1.25	0.33	0.98
4	7.74	0.35	7.86	1.43	2.84	1.16	0.22	0.13
5	7.83	0.41	8.56	1.26	1.98	0.86	0.25	0.13
6	8.02	0.51	8.88	1.55	1.49	0.70	0.20	0.30
7	7.90	0.35	8.17	1.13	2.16	0.73	0.18	0.09
8	7.83	0.37	8.84	1.66	1.99	0.96	0.14	0.11
9	7.99	0.35	8.93	1.37	1.97	0.47	0.12	0.05
10	7.28	0.45	7.21	2.02	2.44	0.85	0.53	0.42
11	7.90	0.36	9.05	1.69	2.57	0.94	0.27	0.11
12	7.81	0.38	7.91	1.86	2.69	0.97	0.65	2.52
13	7.51	0.27	7.76	1.35	2.42	0.59	0.22	0.20
14	6.94	0.49	6.80	1.79	2.36	0.91	0.97	1.38
15	7.52	0.21	7.77	1.19	2.44	0.43	0.21	0.07
16	7.85	0.31	7.43	1.61	2.29	0.71	0.19	0.13
17	7.64	0.36	8.35	1.75	2.55	1.28	0.24	0.12

Fig 3 shows the weekly probability distribution functions (PDFs) for regional mean concentrations (the average value of water quality at 17 stations) between 2004 and 2015 for the three time periods: 2004–2007 (black curve), 2008–2011 (green curve), and 2012–2015 (red curve). According to the changes can be inferred through the shift and shape of the curves between three time periods from the Figure, we can see that there has been a noticeable decrease in COD_Mn_ and NH_3_-N over the period (the PDF curve shifted from right (black curve) to left (red curve)), suggesting improvement overall in Yangtze River Basin water quality, especially over the 2012–2015 period; a tendency towards smaller pH-values was also found in recent years.

Overall water quality improvement is shown based on an evaluation of 17 environmental stations, which differs slightly from the State of Environment Report of China [44] indicating Grade I-III water increased slightly from 88.1% in 2014 to 89.4% in 2015, and Grade V+ water remained flat at 3.1%. However, the report agreed in that it showed overall improvement in the situation of the Yangtze River in recent years.

### Seasonal Mann Kendall trends

**Fig 4:**
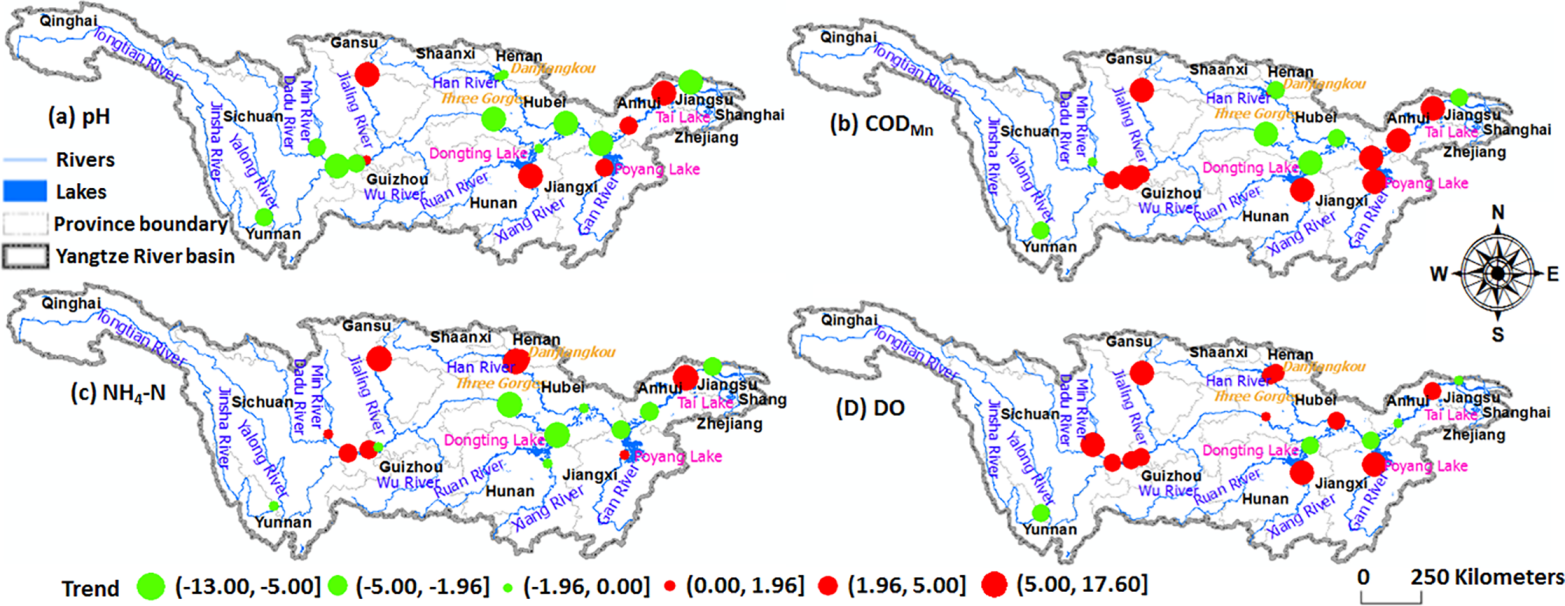
Spatial distribution of seasonal Mann-Kendall trends (the value of Z) at 17 stations in the Yangtze River basin, between 2004 and 2015. (a) pH, (**b**) COD_Mn_ (mg/L), (**c**) NH_3_-N (mg/L), and (**d**) DO (mg/L).

Fig 4 and Table 3 show seasonal trends (the value of the test statistic *Z*) at 17 stations in the Yangtze River basin from 2004 to 2015. pH tended to decrease at 11 stations (approximately 65%, 8 stations with significance at 95% confidence), which are mainly located on the upper and middle of the Yangtze River basin (Fig 4A). Total 10 stations (among them, 9 stations with significance at 95% confidence) exhibited a positive trend in COD_Mn_ concentration, mainly occurring on major tributaries (e.g., station 10 on Xiang River, station 14 on Gan River) and the lower reach of the trunk stream (Fig 4B). For NH_4_-N concentration, about half of the stations showed decreasing trends, most of which are distributed on the middle and lower reaches of the trunk stream (Fig 4C). Increasing seasonal trends were found at 12 stations for DO concentration (Fig 4D).

**Table 3:** Seasonal trends (the value of Z) of stations with positive or negative trends for water quality parameters in the Yangtze River basin during 2004–2015.

ID	Range in z scores	Showing positive trend	Showing significant positive trend	Showing negative trend	Showing significant negative trend
pH	-10.3–11.6	6	5	11	8
COD_Mn_	-12.9–11.3	10	9	7	6
NH_4_-N	-10.8–15.3	8	6	9	5
DO	-3.1–17.6	12	11	5	3

**Fig 5:**
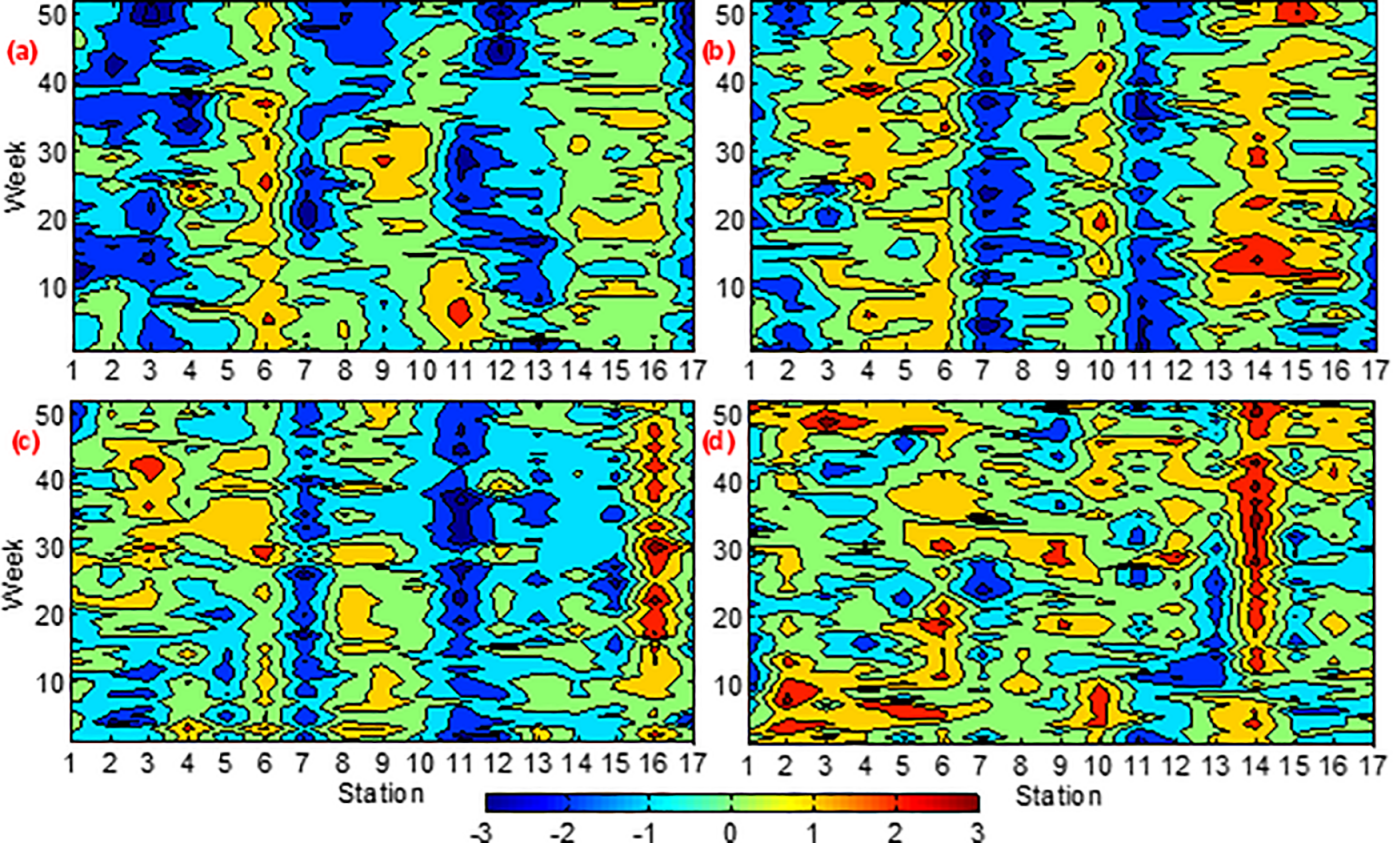
Mann-Kendall trends (the value of Z) for 52 weekly water qualityat 17 stations in the Yangtze River basin, between 2004 and 2015. (a) pH, (**b**) COD_Mn_ (mg/L), (**c**) NH_3_-N (mg/L), and (**d**) DO (mg/L).

Fig 5 shows the Mann-Kendall trends (the value of Z) for 52 weeks at 17 stations in the Yangtze River basin, between 2004 and 2015, showing increasing long-term trends. From the figure, we can see that decreasing trends were found in COD_Mn_ and NH_4_-N concentrations at station 7 and station 11 over 52 weeks, while increasing trends in four water quality parameters at station 6 over 52 weeks, in NH_3_-N concentration at station 16 and in COD_Mn_ and DO concentrations at station 10 and station 14 over the time.

### Time-series decomposition results

#### Long-term trends

**Fig 6:**
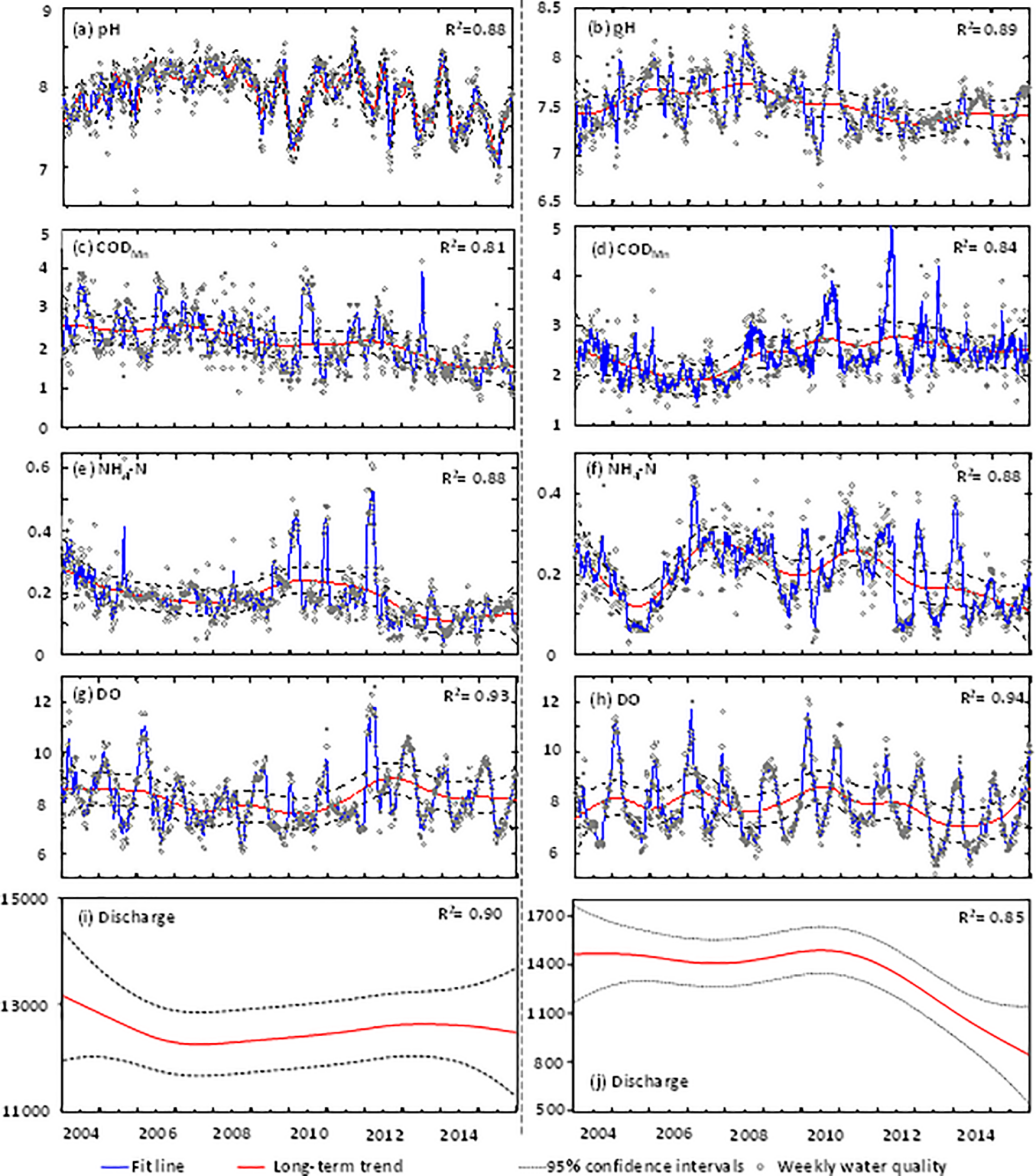
Comparison of the long-term trend of pH, DO, COD_Mn_, NH_4_-N at environmental stations 7 (left) and 13 (right). The last two sub-graphs are for trends of river discharges at Yichang and Hukou respectively. The coefficients of determination (R^2^) between the weekly data and the DHR model are given in each panel.

Results of the DRH analysis, conducted based on two representative environmental stations, are illustrated in Fig 6. Significant long-term trends at these two environmental stations were identified for pH, DO, COD_Mn_, and NH_3_-N. The R^2^ values for them ranged from 0.81 for COD_Mn_ at Nanjinguan station to 0.94 for DO at Hexishuichang station, indicating good performance. Additionally, pH showed very clear seasonal variation at both stations, but the periodic disturbance at Nanjinguan station was more frequent (Fig 6A and 6B). It is possible that the operation of the three Gorges Reservoir (TGR) enhanced pH fluctuations at Nanjinguan station. For example, Wang et al. (2015) showed that the operation of releasing bottom water for power generation decreased the pH downstream from the dam because of the change of [CO_2_] / [CO_3_^2−^] ratios [45]. Fig 6A shows that pH increased from 7.8 in January 2004 to 8.4 in July 2007 with a strong seasonal signal, then declined to 7.2 around January 2009 and then reached a new high in August 2011 and finally declined. At Hexishuichang station, pH increased from 7 in January 2004 to 8.3 in July 2008 and then decreased until June 2013 and finally remained at the same level.

As shown in Fig 6C, significant long-term declines were found in stream water COD_Mn_ concentrations at Nanjingguan station, the value of which was about 2.5 mg/l in January 2004 and finally decreased to1.5 mg/l in December 2015. One reason might be the attenuation of the TGR, with nutrient and sediment removal rates in reservoirs significantly affecting transport in river systems [46]. Moreover, the impact of the TGR might be unstable because the conditions in the TGR have changed (e.g., operating rules or water supply) during the time period. The establishment of the TGR has three engineering phases including 1992–1997, 1998–2003 and 2003–2009. Also, operating rules have been adjusted for different flooding situations. By comparison, COD_Mn_ at Hexishuichang station initially decreased to around 1.8 mg/l in February 2007, and then experienced two peaks (August 2010 and June 2012), and finally plateaued with only slight variation over the remaining years (Fig 6D).

Clear seasonal variability was found in NH_4_-N concentrations at both stations, and both of them finally decreased from January 2004 to December 2015 (Fig 6E and 6F). NH_4_-N at Nanjinguan station experienced one peak (in June 2010) and then exhibited only slight variability from November 2013, while Hexhishuichang showed three peaks (two big peaks in August 2007 and July 2011, and a smaller one in 2014). At Nanjinguan station, the long-term decreasing trend for COD_Mn_ generally parallels the trend in NH_4_-N, with a significant Pearson’s Correlation coefficient between the COD_Mn_ and NH_4_-N trends (Pearson's r = 0.62, p<0.01).

Fig 6G shows that DO concentrations at Nanjingguan station decreased slightly from January 2004 to June 2010, then increased dramatically around July 2012, and finally decreased in December 2015. In comparison, after three step-wise increases, DO concentrations at Hexishuichang station initially increased and peaked in May 2010, and then decreased to 6 mg/l in July 2014, and finally increased to 10 mg/l in December 2015.

Discharge at Yichang station initially decreased by February 2007, and then varied only slightly over the remaining period of record (Fig 6I). There are no similar overall trends for pH, DO, COD_Mn_, or NH_4_-N concentrations at this station. However, during the period from January 2007- February 2014, a similar decreasing trend was found for DO, COD_Mn_, and NH_4_-N. A slight variability over January 2004—February 2011 and a marked decline from March 2011 were found at Hukou station (Fig 6J), and similarly there was no overall trend in pH, DO, COD_Mn_, or NH_4_-N concentrations.

#### Seasonality

Initial review of weekly time-series plots (Fig 6) indicated a seasonal pattern for pollutants such as pH. Through DHR analysis, seasonal cycles of varying strength were extracted for all pollutant time-series (Table 4 and S3 Fig). Table 4 shows that the strongest seasonal cycle was identified for NH_3_-N at Nanjinguan station (50.00%), while the weakest was for pH at Nanjinguan station (9.55%). Seasonal patterns in pH had small phases at both stations, with concentration maxima in winter, and minima in summer, which is consistent with previous work demonstrating that pH in June–August was slightly lower than in January–March in the middle Yangtze River [47]. The result was similar with other study sites. For example, Zeb et al. indicated that pH in winter was higher than in summer in Khyber Pakhtunkhawa (KPK) province of Pakistan [48].

**Table 4:** Seasonality analysis: The results of the DHR seasonal cycle extracted from the weekly time series (see the main text for more detail on how the seasonality analysis was carried out).

Station	Parameter	Figure	Peak	Minimum	Strength*	Amplitude	Phase
Nanjinguan	pH	Fig 6A	December	June	9.55%	Constant	Constant
	COD_Mn_		June/July	December-February	30.00%	Variations	Constant
	NH_4_-N	Fig 6C	February-April	July-October	50.00%	Variations	Constant
	DO		January-March	June-September	48.39%	Increases	Variations
Hukou	pH	Fig 6B	December	June	15.96%	Constant	Constant
	COD_Mn_		March-May	July-September	18.20%	Variations	Variations
	NH_4_-N	Fig 6D	December-April	May-August	13.20%	Constant	Variations
	DO		January-March	June-August	35.29%	Constant	Constant

* The strength of the seasonal cycle has been taken as the percentage of variability within the dataset (the range of concentrations recorded) explained by the maximum amplitude of the identified seasonal cycle.

COD_Mn_ at both stations exhibited a seasonal cycle with variability in amplitude throughout the study period. Concentration peaks occurred in summer and minima in winter at Nanjinguan station, and peaks in spring and minima in the end of summer and start of autumn at Hexishuichang station. Generally, NH_4_-N and DO exhibited similar seasonal cycles at both stations. The crest and trough of DO reflected higher values in winter and lower values in summer, which agrees with results from [49]. DO concentration tended to track river temperature, with high concentrations in winter and early spring (times of lower water temperature), and low concentrations in summer and fall (times of higher water temperature).

Similar and clear seasonal cycles of varying strength were found for discharge at both stations (S3E and S3F Fig), with discharge maxima in summer, and minima in winter, reflecting seasonal precipitation (wet condition from May to October and dry condition from December to April) influenced by both East and South Asian monsoon activities exhibited in the Yangtze River basin. Discharge patterns mirror pH in seasonality, although the amplitude of discharge was variable, especially at Hukou station.

## Discussions

**Fig 7:**
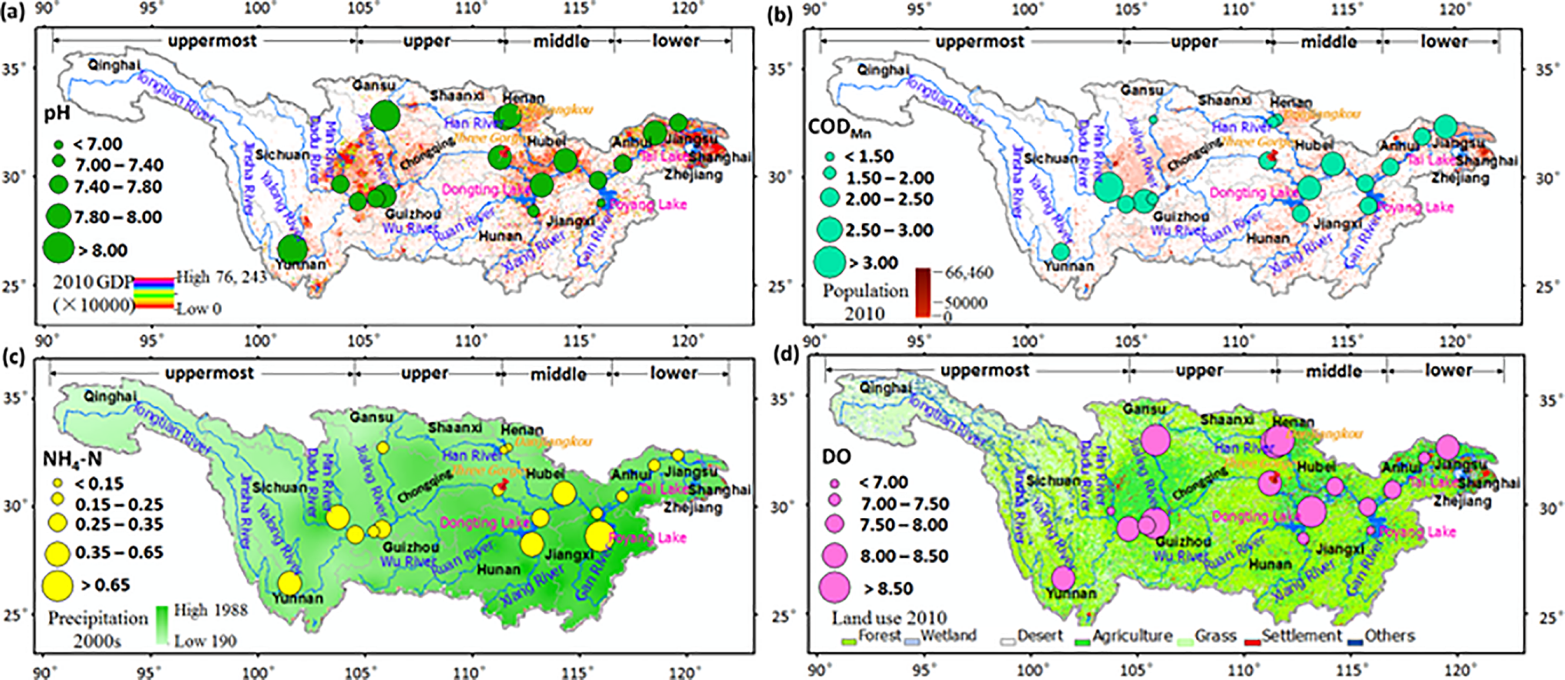
Spatial distributions of mean concentrationsat 17 stations, with GDP of 2010 (Chinese yuan /km^2^), population of 2010 (person/km^2^), mean precipitation between 2001 and 2010 (mm), and land use of 2010. (a) pH, (**b**) COD_Mn_ (mg/L), (**c**) NH_3_-N (mg/L), and (**d**) DO (mg/L).

**Fig 8:**
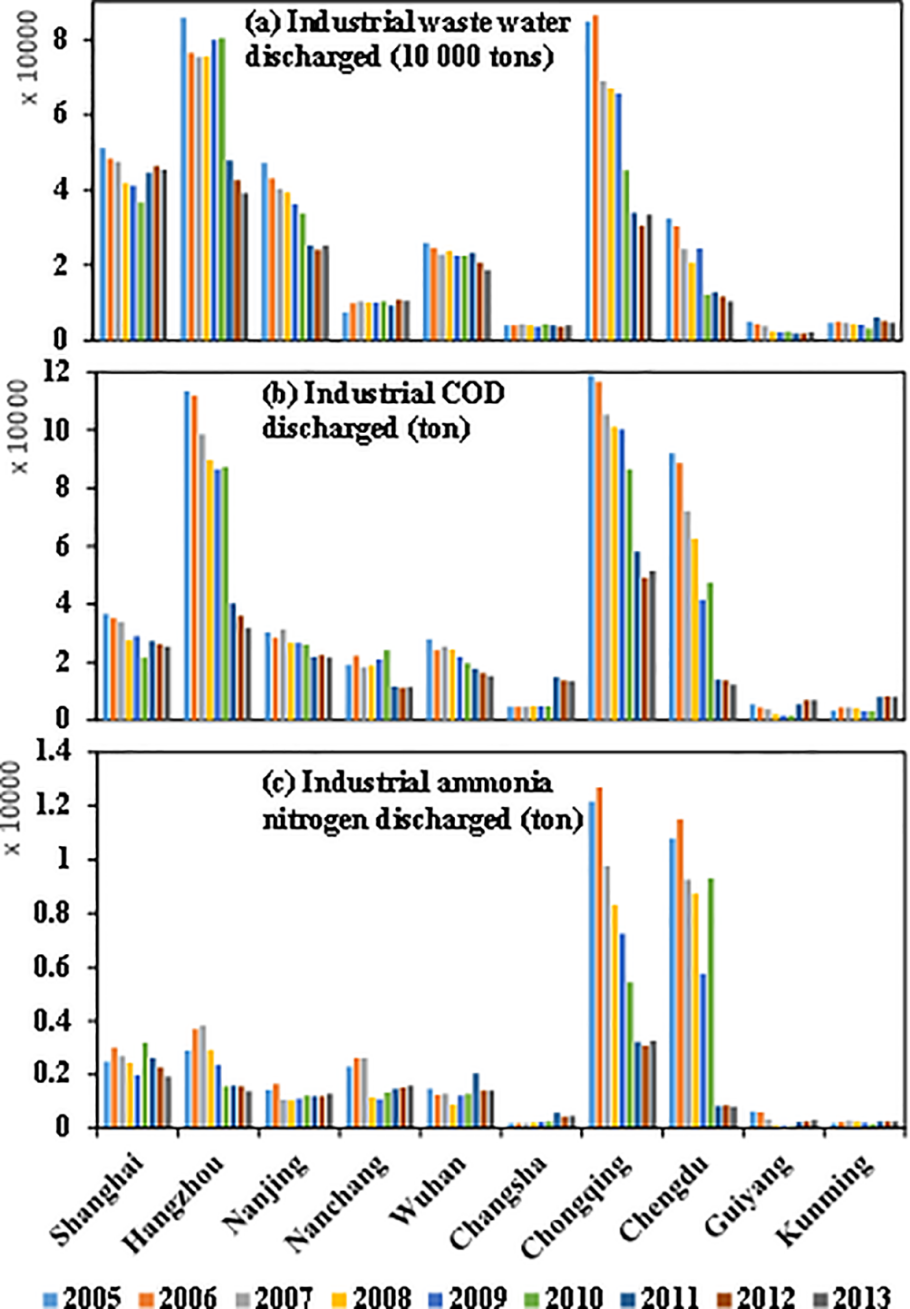
Main pollutant emission in industrial waste water in main cities from 2005 to 2013.

Rapid economic development and increasing population have brought significant changes in land cover and placed stress on water quantity and quality within the Yangtze River basin. Spatial distribution of mean concentration at each station, with GDP of 2010, population of 2010, mean precipitation between 2001 and 2010, gross domestic product (GDP), and land use of 2010, are shown in Fig 7. The Figure shows that GDP and population is concentrated in big cities such as the Chengdu-Chongqing Economic Zone along the Upper Yangtze River, the Wuhan-Changsha-Nanchang Economic Zone along the Middle Yangtze River, and the Yangtze River Delta Economic Zone (e.g., Shanghai, Hangzhou, and Nanjing) along the Lower Yangtze River. Water quality in these area (e.g., stations 2–4, 10, 14, and 17) was relatively poor because of pollutants such as NH_4_-N and COD_Mn_ associated with industrial activities and sanitary sewage [50, 51]. Pollutants in industrial and household waste-water discharges were high in these cities (Fig 8 and S4 Fig), especially in Shanghai, Hangzhou, Chongqing and Chengdu. Moreover, the household waste-water discharged increased between 2011 and 2013 as people migrated to cities from the country [52]. Regional high pollution may be a consequence of drainage from municipal sewage, agricultural wastewater, and livestock production facilities. Water quality is strongly affected by land-use through changes of pollutant input and the water cycle. On the one hand, with the growth of agricultural and settlement land, point source pollution and nonpoint source pollution are likely to increase because of incremental sewage treatment plants and additional use of agricultural fertilizer, like other countries [53]. On the other hand, land use may accelerate pollutants transport by changing the form of runoff. For example, most rain that falls on a parking lot runs off immediately [54], often draining into storm sewers with pollutants that transport it to a stream or ditch without filtration.

In addition, industrial waste-water discharge was nearly unchanged in Nanchang and Changsha between 2005 and 2013, but industrial pollutants and ammonia nitrogen discharge have increased, especially in the last three years (Fig 8). These pollutants entered the Xiang and Gan Rivers, possibly explaining the high concentration and increasing trends at stations 10 and 14. Yan et al. indicated that agricultural activities, industrial and domestic wastewater have caused increasing loads of nitrogen to be discharged into the Yangtze River between 1970 and 2003 [55]. In general, water quality at stations on northern tributaries (including 6, 8 and 9) were better than southern tributaries (e.g., station 10 on the Xiang River and station 14 on the Gan River), and NH_4_-N at stations on the trunk stream were relatively low. Meanwhile, heavy metals and nitrogen are the major pollutants in the lower Xiang [56, 57] and Gan Rivers [58]. Although these changes are likely to contribute to deteriorating water quality, enormous effort has been made in recent decades in China including an ever-improving legal system and the popularity of sewage treatment plants, leading to improving water quality.

Finally, Fig 4 and Table 3 show that there has been a measurable decrease in pollutants before flowing into dams and reservoirs and after flowing from them. For example, water quality after the dams is improving than before the dams; mean NH_4_-N at stations 10 and 14 were 0.53 mg/l and 0.65 mg/l respectively, but dramatically decreased to 0.27 mg/l and 0.20 mg/l at stations 11 and 15 after flowing from Dongting and Poyang Lakes. Possibly, high runoff from other tributaries with low pollutant concentration may dilute outflows from lakes and reservoirs and dams are positively influencing the retention of pollutants along the river. Additionally, attenuation of pollutant concentrations due to lakes is another well-established explanation for the data [59], especially under some of appropriate planning and contamination monitoring policies.

## Conclusions

This study characterizes the spatio-temporal distribution, long-term trend, and seasonality of water quality in the Yangtze River basin using statistic methods and time-series decomposition. The used dataset were weekly water quality data (pH, COD, NH_4_-N, and DO) from 17 environmental stations for the period January 2004 through December 2015. The weekly water quality data allowed analysis of long-term trends and provided new insights into the changing amplitude and phase of the seasonality of the pollutants within the Yangtze River basin. Water quality gradually improved during this time period in the Yangtze River basin, but regional differences are still obvious. For example, high ammonia nitrogen pollution can be seen in the Xiang and Gan River basins because of high pollutants from industrial activities and sanitary sewage around these rivers. In addition, significant seasonal trends were identified in weekly pH, DO, COD_Mn_, and NH_4_-N concentration over the 2004–2015 period, and seasonal cycles of varying strength were extracted for pollutant time-series, suggesting the seasonal cycles of water quality in the Yangtze River basin. All these results could be helpful for fully understanding the seasonal trends of long-term water quality in the Yangtze River basin, which can provide essential information for effectively controlling water pollution and managing water resources.

## Supporting information

S1 Fig Changes of number of different grades of river water based on 17 stations in the Yangtze River basin from 2004 to 2015. (TIF)

S2 Fig Boxplots illustrating distribution of water quality at 17 stations from 2004 to 2015 in the Yangtze River basin. Red lines are the mediums. (a) pH, (**b**) COD_Mn_ (mg/L), (**c**) NH_3_-N (mg/L), and (**d**) DO (mg/L).
(TIF)

S3 Fig Seasonal cycles extracted from the weekly pH, DO, COD_Mn_, NH_4_-N at environmental stations 7 (left) and 13 (right). The last two sub-graphs are for seasonal cycles of river discharges at Yichang and Hukou respectively.
(TIF)

S4 Fig Main pollutant emission in household waste water in main cities from 2005 to 2013. (TIF)

S1 Table Environmental quality standards for surface water(mg/L) (GB3838 2002a). (DOCX)
