# Supplementary figures and images for: Identification of long-term trends and seasonality in high-frequency water quality data from the Yangtze River basin, China
Source: PLoS One. 2018 Feb 21;13(2):e0188889. doi: 10.1371/journal.pone.0188889 (PMC5821306; doi:10.1371/journal.pone.0188889)

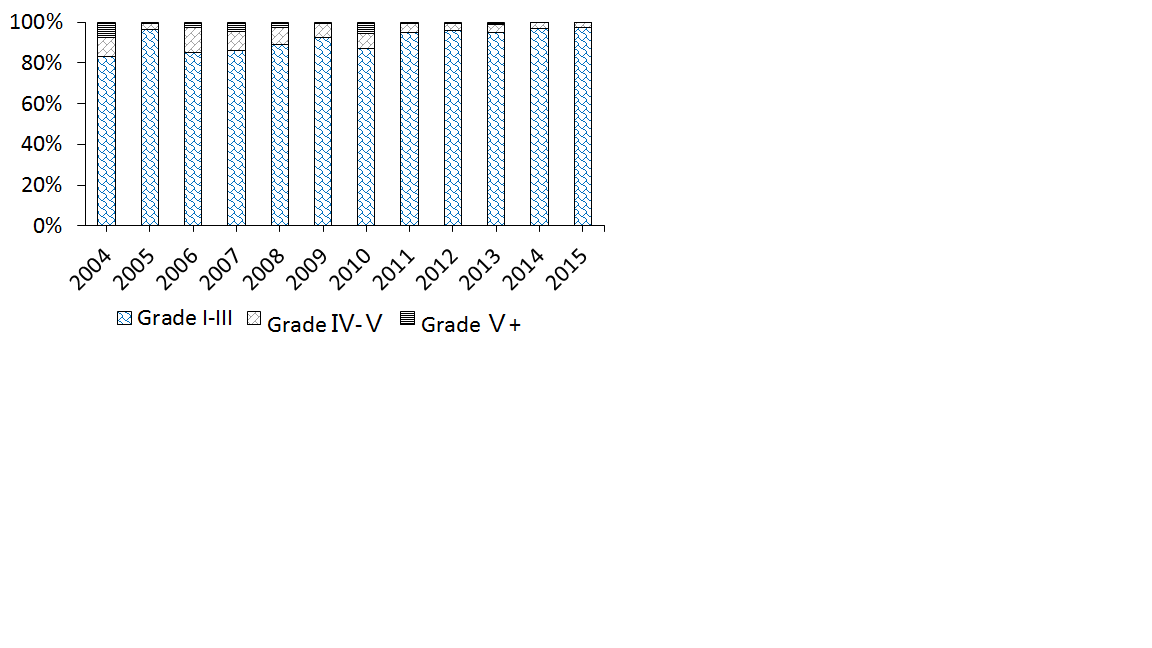

Supplement: S1 Fig — (TIF) [file pone.0188889.s001.tif]

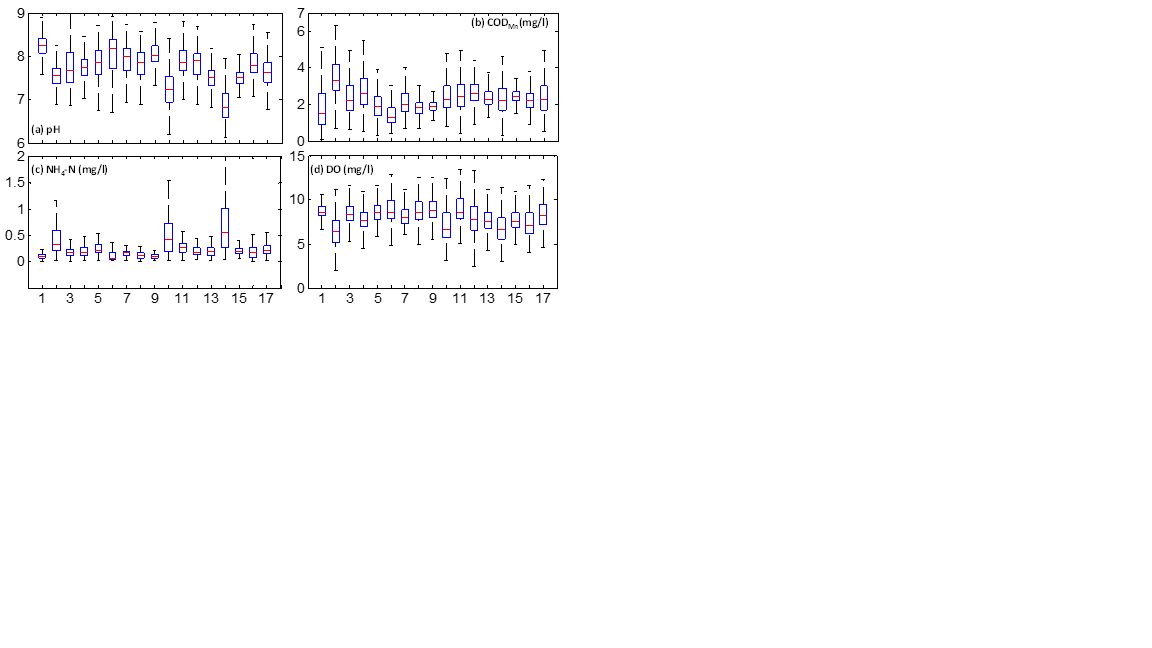

Supplement: S2 Fig — (a) pH, (b) CODMn (mg/L), (c) NH3-N (mg/L), and (d) DO (mg/L). (TIF) [file pone.0188889.s002.tif]

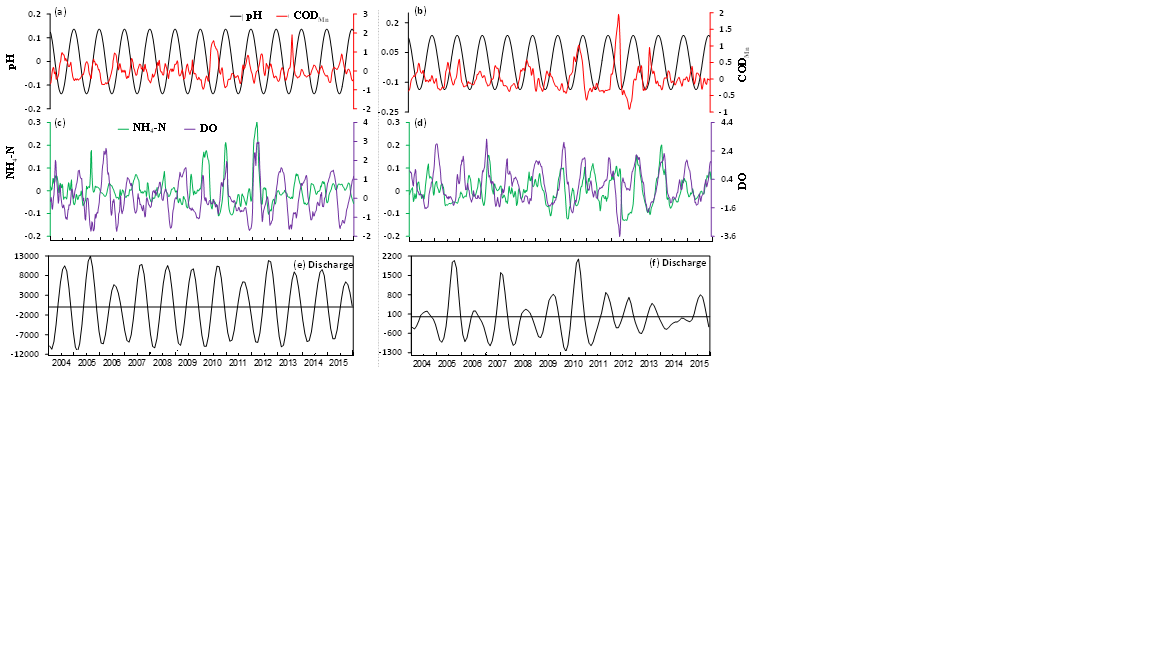

Supplement: S3 Fig — The last two sub-graphs are for seasonal cycles of river discharges at Yichang and Hukou respectively. (TIF) [file pone.0188889.s003.tif]

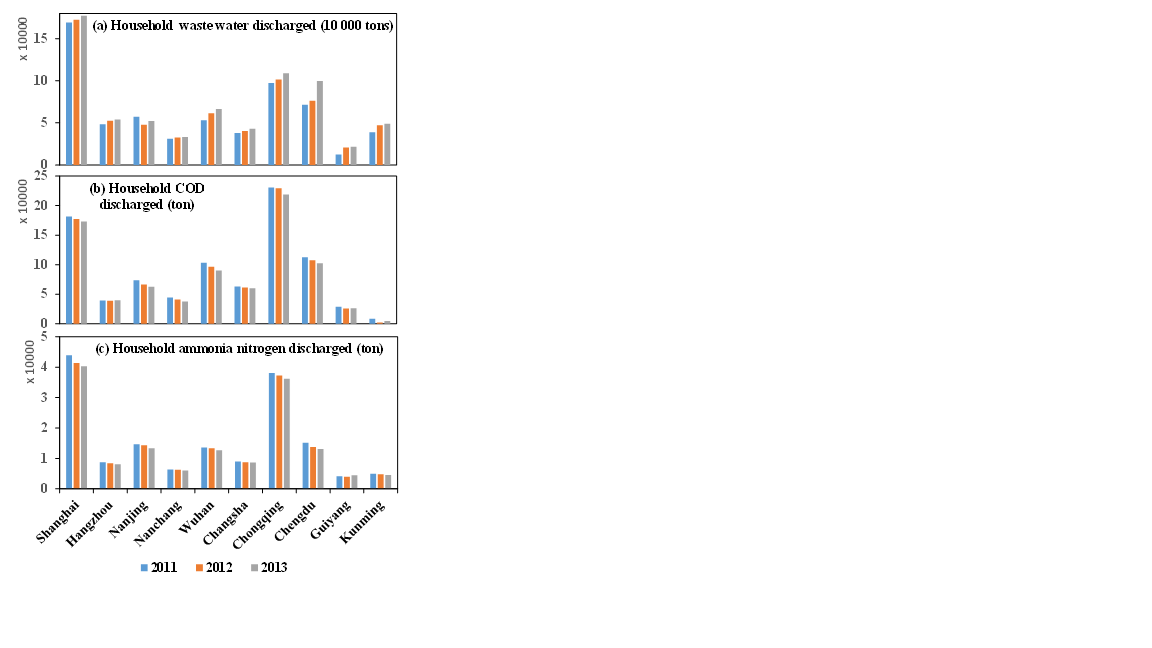

Supplement: S4 Fig — (TIF) [file pone.0188889.s004.tif]
