# Supplementary material for: Identification of long-term trends and seasonality in high-frequency water quality data from the Yangtze River basin, China
Source: PLoS One. 2018 Feb 21;13(2):e0188889. doi: 10.1371/journal.pone.0188889 (PMC5821306; doi:10.1371/journal.pone.0188889)
Supplement: S1 Table — (DOCX) [file pone.0188889.s005.docx]

**S1 Table** Environmental quality standards for surface water(mg/L) ([GB3838 2002a](#_ENREF_22))

| number | parameters |  | TypeⅠ | TypeⅡ | Type Ⅲ | Type Ⅳ | Type Ⅴ |
| --- | --- | --- | --- | --- | --- | --- | --- |
| 1 | Water  Temperature  (℃) |  | Man-made water temperature change should be limited :  Average weekly maximum temperature rise≤1  Average weekly maximum temperature drop ≤2 | | | | |
| 2 | pH |  | 6---9 | | | | |
| 3 | DO | ≥ | saturation ratio 90%（or7.5） | 6 | 5 | 3 | 2 |
| 4 | COD_Mn_ | ≤ | 2 | 4 | 6 | 10 | 15 |
| 5 | COD_Cr_ | ≤ | 15 | 15 | 20 | 30 | 40 |
| 6 | BOD_5_ | ≤ | 3 | 3 | 4 | 6 | 10 |
| 7 | NH_4_-N | ≤ | 0.15 | 0.5 | 1.0 | 1.5 | 2.0 |
| 8 | TP | ≤ | 0.02 （Lake=0.01） | 0.1 （Lake=0.025） | 0.2 （Lake=0.05） | 0.3 （Lake=0.1） | 0.4 （Lake=0.2） |
| 9 | TN | ≤ | 0.2 | 0.5 | 1.0 | 1.5 | 2.0 |
| 10 | Cu | ≤ | 0.01 | 1.0 | 1.0 | 1.0 | 1.0 |
| 11 | Zn | ≤ | 0.05 | 1.0 | 1.0 | 2.0 | 2.0 |
| 12 | Fˉ | ≤ | 1.0 | 1.0 | 1.0 | 1.5 | 1.5 |
| 13 | Se | ≤ | 0.01 | 0.01 | 0.01 | 0.02 | 0.02 |
| 14 | As | ≤ | 0.05 | 0.05 | 0.05 | 0.1 | 0.1 |
| 15 | Hg | ≤ | 0.00005 | 0.00005 | 0.0001 | 0.001 | 0.001 |
| 16 | Cd | ≤ | 0.001 | 0.005 | 0.005 | 0.005 | 0.01 |
| 17 | Cr^6+^ | ≤ | 0.01 | 0.05 | 0.05 | 0.05 | 0.1 |
| 18 | Pb | ≤ | 0.01 | 0.01 | 0.05 | 0.05 | 0.1 |
| 19 | cyanide | ≤ | 0.005 | 0.05 | 0.2 | 0.2 | 0.2 |
| 20 | volatile phenol | ≤ | 0.002 | 0.002 | 0.005 | 0.01 | 0.1 |
| 21 | petroleum | ≤ | 0.05 | 0.05 | 0.05 | 0.5 | 1.0 |
| 22 | anionic surfactant | ≤ | 0.2 | 0.2 | 0.2 | 0.3 | 0.3 |
| 23 | Sulfide | ≤ | 0.05 | 0.1 | 0.05 | 0.5 | 1.0 |
| 24 | [fecal coliform](http://dict.cn/fecal%20coliform)（unit/L） | ≤ | 200 | 2000 | 10000 | 20000 | 40000 |

*Note: This surface water quality standard is an authorized guideline now available in China (GB3838-2002), in which water quality standard Type I refers to water quality comparable to the source water or national nature reserve water, Types II and III refers to the protection zones mainly used in the centralized surface drinking water source, Types IV and V represent water that is no longer fit for drinking but still suitable for industrial and agricultural usage, and water quality worse than Type V is severely detrimental to aquatic ecosystem health.*
